# Character

**Published:** 1910-07

**Authors:** 


### Abstracts and Selections.

### Character.

A man's whole schooling is worthless if it has not made him reverent and gentle. All the learning that can be soaked into the human hide isn't as useful as simple human goodness. He is a failure who has not discovered that it is a bigger thing to lean against a man than to lean against a bank vault. No student deserves a diploma unless his course of study has given him a conquering soul, a mellow heart, a mind passionate for helpfulness and a growing reverence which makes him a worshiper at work and play; which makes him kneel before all women; which makes him gentle before all men and unafraid before himself; which makes all his thoughts and acts incense upon an altar of white character.—*Exchange*.

